# An Unusual Case of Vasospastic Angina Resulting in Multiple Episodes of Cardiac Arrest

**DOI:** 10.7759/cureus.51944

**Published:** 2024-01-09

**Authors:** Francisco d'Orey, Joao Nuno Patricio, Maria Inês Ribeiro, Hugo Côrte-Real

**Affiliations:** 1 Intensive Care Unit, Hospital Garcia de Orta, Almada, PRT; 2 Intensive Care Unit, Hospital Beatriz Ângelo, Loures, PRT; 3 Critical Care, Hospital Garcia de Orta, Almada, PRT; 4 Intensive Care Department, Hospital de Santa Maria, Lisbon, PRT

**Keywords:** vasospastic angina, coronary vessel anomaly, vasospastic disorders, prinzmetal’s angina, in hospital cardiac arrest

## Abstract

Coronary vasospasm is a well-recognized cause of angina (also known as Prinzmetal angina) and a common cause of admissions to the emergency department and coronary intensive care units. It is however an uncommon cause of cardiac arrest. We describe a patient with multiple episodes of chest pain followed by cardiac arrest in pulseless electrical activity (PEA) due to coronary vasospasm. Telemetry and electrocardiography showed ST-segment elevation followed by PEA. Each event was short-lived and resolved after a maximum of six minutes of advanced life support measures. The patient was started on treatment with a dihydropyridine calcium channel blocker (CCB) and nitroglycerin patch with no further episodes recorded to date.

## Introduction

Coronary vasospasm is a well-recognized cause of angina (also known as Prinzmetal angina) and a common cause of admissions to the emergency department and coronary intensive care units. Vasospastic angina was first described by Prinzmetal and colleagues in 1959 as a syndrome of ischemic pain that occurred at rest, accompanied by ST-segment elevation [1]. It can lead to a number of clinical presentations including syncope, myocardial infarction, arrhythmias (in rare cases, ventricular fibrillation (VF) and tachycardia (VT)), or even no symptoms. This phenomenon is heterogeneous and can occur in patients with or without coronary atherosclerosis, being either focal or diffuse and affecting epicardial or microvasculature coronary arteries [2,3]. Precipitating factors for vasospasm include smoking, alcohol consumption, cocaine use, physical and emotional stress, Valsalva maneuver, hypercholesterolemia, hyperventilation, and sympathomimetic medications [4,5]

The pathogenesis is not yet fully understood but there are some pathogenic mechanisms that can contribute to it such as vascular smooth muscle cell hyperreactivity, endothelial dysfunction, magnesium deficiency, low-grade inflammation, altered autonomic nervous system response, and oxidative stress [5,6].

Coronary vasospasm is, however, an uncommon cause of cardiac arrest. We describe a patient with multiple episodes of chest pain followed by cardiac arrest in pulseless electrical activity (PEA) due to coronary vasospasm.

## Case presentation

**Figure 1:**
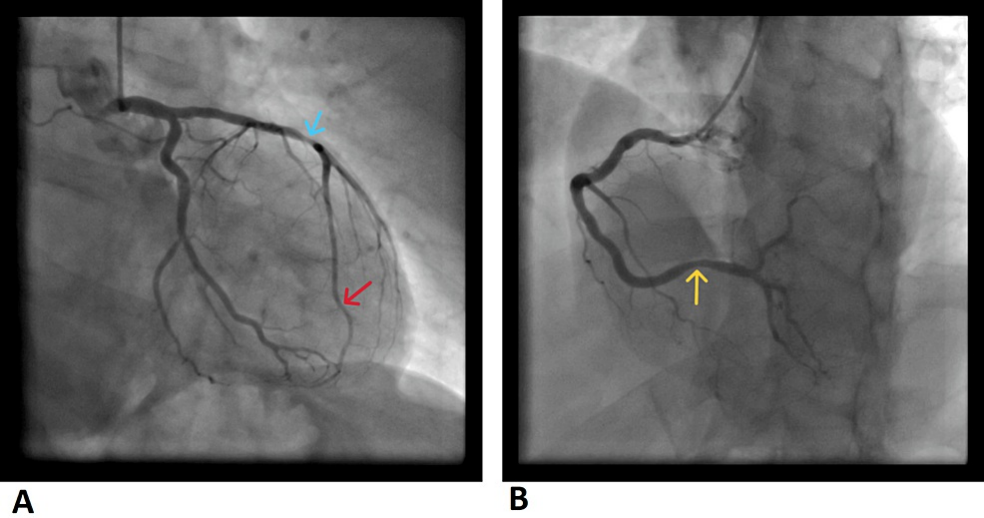
Coronary angiography after first cardiac arrest episode. (A) Left coronary artery. Blue arrow shows the LAD segment with 50% stenosis, TIMI coronary flow grade 3, and the red arrow shows the distal LAD with 90% stenosis segment, stent inserted, TIMI 3; (B) Right coronary artery. Yellow arrow shows 1-30% stenosis, TIMI 3. All stenosis had little or no calcium. LAD: left anterior descending; TIMI: thrombolysis in myocardial Infarction

A 53-year-old man with no relevant past medical or family history and no known cardiovascular risk factors, presented to the angiography unit after an episode of oppressive chest pain associated with cardiac arrest in PEA, during a routine ophthalmology consultation. He fully recovered after six minutes of advanced life support (ALS), which included the administration of two doses of 1mg of adrenaline (as per ALS guidelines). He underwent urgent coronary angiography aided by optical coherence tomography (OCT) and a drug-eluting stent was placed in the distal segment of the left anterior descending artery, which demonstrated significant stenosis (Figure 1). During the period that followed the cardiac arrest and angiography, he remained in sinus rhythm with normal vital signs.

He had had an episode of chest pain associated with syncope three months previously which had been thoroughly investigated: echocardiogram, coronary angiography, computed tomography (CT) pulmonary-angiogram, and CT head, none showing any relevant abnormalities.

**Figure 2:**
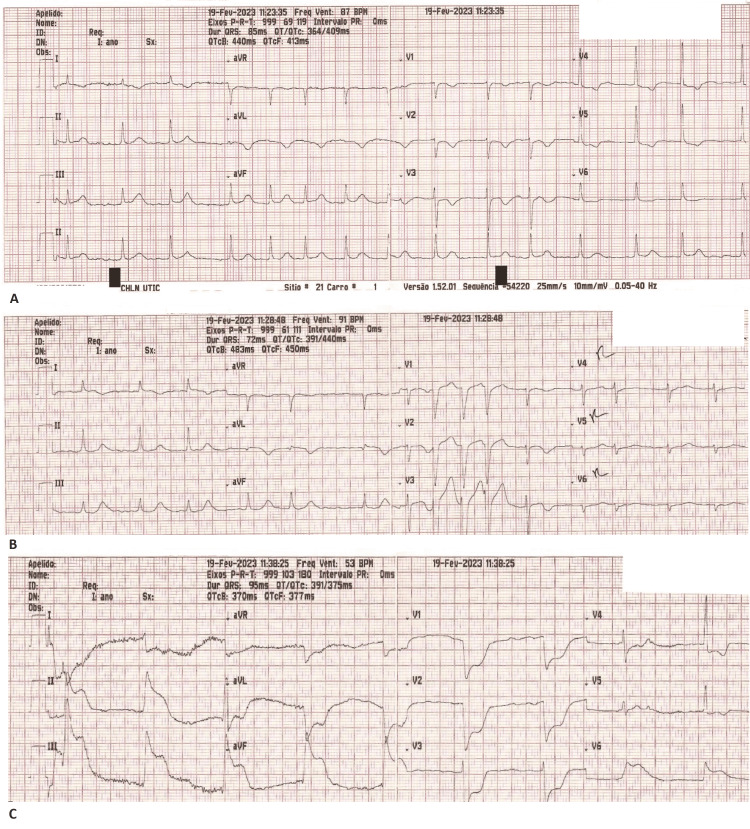
ECG sequence during the second episode of chest pain and cardiac arrest. (A) ECG taken at the beginning of chest pain and patient was noted to be in atrial fibrillation. There is also T wave inversion in V2-V6 and leads I and aVL and ST depression V4-V6 suggesting ischemia in the LCA territory; (B) ECG recorded five minutes after the first, showing non-sustained monomorphic ventricular tachycardia (three complexes V1-V3), ST segment elevation in leads I and aVL; (C) ECG taken 10 minutes after the second, showing complete AV node block, ST segment elevation in inferior leads with reciprocal changes in lateral and anterior leads suggesting ischemia in the RCA territory. A few seconds after this ECG was recorded, the patient went into cardiac arrest in PEA. AV: atrioventricular; RCA: right coronary artery; PEA: pulseless electric activity

The patient was admitted to the coronary intensive care unit and during his stay he had two further episodes of sudden chest pain at rest, which progressed to cardiac arrest, resolving after four minutes of ALS each (including the administration of 1 mg of adrenaline intravenously). During the first episode, he was initially conscious, in atrial fibrillation, quickly progressing to complete heart block and cardiac arrest in PEA. ECGs were recorded throughout the episode (Figure 2). The second episode started during the removal of a peripheral cannula and quickly progressed into PEA cardiac arrest. On both occasions, telemetry showed ST-segment elevation. Echocardiography post-arrest showed generalized hypokinesia of the left ventricle and urgent coronary angiography showed no new coronary lesions.

Given the clinical picture, ECGs, and coronary angiography findings, a likely diagnosis of vasospastic angina was made and the patient was started on nifedipine and nitroglycerin transdermal patch. Since all documented arrests were in a non-shockable rhythm, a decision was made not to implant an implantable cardioverter-defibrillator (ICD). The patient remained under observation for another week with no further episodes. 

Before discharge, a cardiac magnetic resonance (CMR) was done which showed normal heart volumes, normal biventricular function, and a small subendocardial infarction in the anterior apical segment, in the anterior descending territory.

## Discussion

Coronary vasospasm typically presents with acute chest pain, transient ST-segment elevation or depression on ECG, but no relevant changes on coronary angiography. When vasospasm lasts long enough, it can result in myocardial infarction, atrioventricular block, life-threatening arrhythmia (VF and VT), and sudden death. Nonetheless, cardiac arrest due to malignant arrhythmias is rare and results from myocardial ischemia [3]. When the diagnosis is unclear, coronary vasospasm can be provoked via the acetylcholine spasm provocation test [2,3,7].

Interestingly, just before the second cardiac arrest, our patient had ECG changes suggestive of ischemia in the inferior wall (showing ST segment elevation in inferior leads with reciprocal changes in lateral and anterior leads) (Figure 2), suggesting vasospasm of the right coronary artery which did not have any significant pathology in the coronary angiographies done.

The striking feature in this case was the recurrent sudden PEA cardiac arrests in the same patient within the same hospital admission and to our knowledge, there are no reports of similar cases. At least on two occasions, vasospasm occurred during a medical procedure: eye examination in the first and removal of an IV cannula in the second suggesting that possibly vasospasm was secondary to parasympathetic activation. The witnessed clinical picture in each "attack" combined with the ECG changes wore enough evidence of coronary vasospasm and provocation tests were deemed unnecessary.

After the presumptive diagnosis of vasospastic angina following the second cardiac arrest, our patient was started on antispastic treatment with a dihydropyridine calcium channel blocker (CCB) and then also on a nitroglycerin patch after the third episode. This combination therapy proved to be effective with no further episodes recorded to date. Since this patient, in all three episodes, never had a shockable rhythm, it was decided not to insert an ICD for secondary prevention.

## Conclusions

Vasospastic angina is a well-known entity and, although being well described, it remains poorly understood. It is an important differential in the work-up of chest pain or acute coronary syndrome as it can be treated, and if found early, the onset of malignant arrhythmias can be prevented. Our case is quite unique since it describes a patient with multiple episodes of cardiac arrest (in PEA) secondary to coronary vasospasm. Even though it is common practice to insert an ICD for secondary prevention, due to the nature of the arrhythmias in our patient, this was deemed inappropriate and a decision was made not to insert one. He recovered with no major clinical consequences and has remained well with no further episodes of angina.

## References

[1] Prinzmetal M, Kennamer R, Merliss R, Wada T, Bor N (1959). Angina pectoris. I. A variant form of angina pectoris; preliminary report. Am J Med.

[2] Picard F, Sayah N, Spagnoli V, Adjedj J, Varenne O (2019). Vasospastic angina: a literature review of current evidence. Arch Cardiovasc Dis.

[3] Hung MJ, Hu P, Hung MY (2014). Coronary artery spasm: review and update. Int J Med Sci.

[4] Rodríguez-Mañero M, Oloriz T, le Polain de Waroux JB (2018). Long-term prognosis of patients with life-threatening ventricular arrhythmias induced by coronary artery spasm. Europace.

[5] Kim HL, Lee SH, Kim J (2016). Incidence and risk factors associated with hospitalization for variant angina in Korea. Medicine (Baltimore).

[6] Cenko E, Bergami M, Varotti E, Bugiardini R (2018). Vasospastic angina and its relationship with the coronary microcirculation. Curr Pharm Des.

[7] Castelein T, Tavernier R, Muyldermans L (2018). Prinzmetal angina can kill twice. Acta Cardiol.

